# Low-Dose vs. High-Dose Cisplatin: Lessons Learned From 59 Chemoradiotherapy Trials in Head and Neck Cancer

**DOI:** 10.3389/fonc.2019.00086

**Published:** 2019-02-21

**Authors:** Petr Szturz, Kristien Wouters, Naomi Kiyota, Makoto Tahara, Kumar Prabhash, Vanita Noronha, David Adelstein, Dirk Van Gestel, Jan B. Vermorken

**Affiliations:** ^1^Department of Oncology, Lausanne University Hospital (CHUV), Lausanne, Switzerland; ^2^Scientific Coordination and Biostatistics, Antwerp University Hospital, Edegem, Belgium; ^3^Faculty of Medicine and Health Sciences, University of Antwerp, Antwerp, Belgium; ^4^Kobe University Hospital Cancer Center Kobe, Japan; ^5^Department of Head and Neck Medical Oncology, National Cancer Center Hospital East, Kashiwa, Japan; ^6^Department of Medical Oncology, Tata Memorial Hospital, Mumbai, India; ^7^Department of Hematology and Medical Oncology, Taussig Cancer Institute, Cleveland Clinic, Cleveland, OH, United States; ^8^Department of Radiotherapy, Institut Jules Bordet, Université Libre de Bruxelles, Brussels, Belgium; ^9^Department of Medical Oncology, Antwerp University Hospital, Edegem, Belgium

**Keywords:** head and neck cancer, chemoradiotherapy, fractionation, cisplatin, clinical trials, cumulative dose, practice recommendations

## Abstract

In locally advanced squamous cell carcinomas of the head and neck (LA-SCCHN), concurrent chemoradiotherapy is an integral part of multimodality management both in the adjuvant and in the definitive settings. Although de-intensification strategies have been propelled to the forefront of clinical research in human papillomavirus (HPV) positive oropharyngeal cancer, three cycles of 100 mg/m^2^ cisplatin given every 3 weeks concurrently with conventionally fractionated external beam radiotherapy represent a cost-effective and globally accessible treatment option for the majority of LA-SCCHN cases. Based on four large randomized trials, this regimen has become the non-surgical standard of care for cisplatin-eligible patients. Nevertheless, the outcomes in terms of efficacy, toxicity, and compliance have been rather disappointing. Therefore, there is an unmet need to find a better alternative. With limited support from randomized trials, weekly low-dose cisplatin regimens have replaced the standard high-dose schedule at some institutions. Four prospective trials exploring radiotherapy with and without weekly low-dose cisplatin have been published. Two of them were conducted in the 1980s, one of which had a negative outcome, the third study provided insufficient information on toxicity, and the fourth trial had to be prematurely terminated due to poor accrual. Moreover, the findings of two phase III trials comparing the two concurrent cisplatin regimens favored the high-dose protocol. We performed a composite meta-analysis of 59 prospective trials enrolling a total of 5,582 patients. The primary endpoint was overall survival. Reflecting different radiotherapy fractionation schemes and treatment intents, three meta-analyses were carried out, one for postoperative conventional chemoradiotherapy, one for definitive conventional chemoradiotherapy, and one for definitive altered fractionation chemoradiotherapy. In the former two settings, both high- and low-dose regimens yielded similar survival outcomes, thus, the primary objective was not met. When given concurrently with altered fractionation radiotherapy, patients treated with high-dose cisplatin had significantly longer overall survival than those who received low-dose cisplatin. In this article we provide a synthetic view of the results, discuss the issue of cumulative dose, compare two vs. three cycles of high-dose cisplatin, and present our three-step recommendations for use of the current standard of care, high-dose cisplatin, in clinical practice.

## Introduction

In squamous cell carcinomas of the head and neck (SCCHN), the prevailing clinical presentation is a locoregionally advanced (LA) disease stage, for which patients are usually offered a multimodality approach involving chemoradiotherapy (1, 2). With the primary intent to eradicate either macroscopic or microscopic disease, this modality represents a relatively simple, inexpensive, and broadly available treatment option, albeit severe acute and late toxicities pose a substantial burden to the patients (3). Based on four large randomized trials, conventionally fractionated external beam radiotherapy with concurrent administration of three cycles of high-dose cisplatin (100 mg/m^2^) given once every 3 weeks represents the current standard in definitive and adjuvant treatment of LA-SCCHN, as it results in significantly better locoregional control and/or overall survival relative to radiotherapy alone (4–7). Nevertheless, concerns about its toxicity and compliance have made many practicing physicians to opt for alternative regimens. In routine practice, the options for possible treatment modifications are limited by existing guidelines, local logistics, and specific reimbursement policies. Therefore, individualization of chemoradiotherapy protocols in LA-SCCHN usually consists in tweaking radiotherapy parameters in terms of total dose, fractionation, and technique and chemotherapy parameters like the type of systemic agent, its peak dose, dose intensity, cumulative dose, and timing of its delivery. In this scenario, despite a clear lack of convincing evidence from controlled trials, weekly concurrent chemotherapy based on low-dose cisplatin gained broader popularity. The major motivation for this transition has probably been toxicity concerns and logistic reasons (see further in the text).

The lack of clinical data, which would legitimize the use of weekly low-dose cisplatin in chemoradiotherapy protocols of non-nasopharyngeal LA-SCCHN, prompted us to conduct a set of meta-analyses scrutinizing high-dose and low-dose cisplatin regimens. Special care was taken to differentiate between the definitive and adjuvant settings as well as between conventional and altered fractionation radiotherapy. The results were published in two full-text papers (8, 9). The primary aim of this article is to provide a synthesis of our major findings, putting them in the context of other relevant publications, to present recommendations for clinical practice, and to open venues for future research.

## Evidence from Controlled Trials

According to the inclusion criteria of our meta-analyses presented below, high-dose cisplatin was defined by a dose of 100 mg/m^2^ given once every 3–4 weeks for a total of three doses if combined with conventional radiotherapy or two doses if combined with altered fractionation radiotherapy. Low-dose cisplatin was defined by a dose not exceeding 50 mg/m^2^ given at weekly intervals for a total of at least six applications in the case of conventional radiotherapy or at least four applications if combined with altered fractionation radiotherapy. Definitive radiotherapy based on conventional fractionation consisted of standard 2 gray (Gy) daily fractions over 7–7.5 weeks with weekend breaks reaching a total dose of about 70 Gy. In the adjuvant setting, 60–66 Gy were given over 6–6.5 weeks. Altered fractionation included hyperfractionation, acceleration, or various combinations thereof, such as concomitant boost technique or simultaneous integrated boost. In the case of hyperfractionation, two to three smaller fractions of 1.1–1.2 Gy per day were given over the same total treatment time. In the case of acceleration, radiotherapy duration was shortened as much as to 5 weeks by extending the number of daily fractions, usually to 6 per week.

With respect to randomized trials comparing one of the two cisplatin-based chemoradiation protocols (high-dose three-weekly or low-dose weekly) vs. radiotherapy only, Tables 1–3 provide a summarizing overview of 9 studies (4–7, 10–17). Concurrently with conventional radiotherapy, a low-dose regimen was explored in 1 and 3 studies in the adjuvant and definitive settings, respectively, whereas the high-dose regimen was studied in 2 and 3 trials, respectively. From this perspective, studies focusing on altered fractionation radiotherapy are lacking. So far, only two trials directly compared weekly with three-weekly cisplatin (Tables 4–6). Another phase II/III trial of the Japanese Clinical Oncology Group (protocol JCOG1008) is ongoing to evaluate the non-inferiority of concurrent chemoradiotherapy with weekly (7 × 40 mg/m^2^) relative to three-weekly cisplatin (3 × 100 mg/m^2^) in the adjuvant setting (20).

**Table 1:** Characteristics of prospective studies comparing chemoradiotherapy with weekly or three-weekly cisplatin vs. radiotherapy alone (4–7, 10–15).

**Concurrent cisplatin regimen**	**Study number, author, year, study design**	**Therapy intent**	**Study arms**	**Inclusion period**	**Number of patients**				**Conventional radiotherapy**		**Concurrent cisplatin**			
					**ITT for the entire study population**	**ITT for cisplatin arm**	**started on cisplatin therapy**	**OPC**	**planned dose to the primary (Gy), technique**	**radiotherapy compliance^**b**^**	**planned schedule**	**planned cumulative dose (mg/m^**2**^)**	**patients receiving all planned cycles**	**patients receiving at least 200 mg/m^**2**^ & at least 2 cycles**
Weekly low-dose	1. Bachaud, 1991, Phase III (10)	Adjuvant	CRT vs. RT alone	1984–1988	88	43	39	7/39 (18%)	65-74, 2D	36/39 & 39/39	7–9 × 50 mg/m^2^	350–450	23/39 (59%)	NR
	2. Sharma, 2010, Phase II randomized (11)	Definitive	CRT vs. RT alone	2003–2005	176	89	77	48/77 (62%)	70, 2D or 3D	49/77 & 71/77	7 × 40 mg/m^2^	280	71/77 (92%)	NR
	3. Quon, 2011, Phase III, E2382 (12)	Definitive	CRT vs. RT alone	1982–1987	371	186	149	37/149 (25%)	70, 2D	NR	7 × 20 mg/m^2^	140	NR	0/149 (0%) & N/A
	4. Ghosh-Laskar, 2016, Phase III (13)	Definitive	CRT vs. RT alone vs. accelerated RT	2000–2007	199	69	65	38/65 (55%)	66-70, 2D	53/65 & 57/65	7–8 × 30 mg/m^2^	210–240	NR	NR
Three-weekly high-dose	5. Cooper, 2004, Phase III, RTOG 9501 (4)	Adjuvant	CRT vs. RT alone	1995–2000	459	228	206	99/206 (48%)	60-66, NR	NR	3 × 100 mg/m^2^	300	125/206 (61%)	NR & 172/206 (84%)
	6. Bernier, 2004, Phase III, EORTC 22931 (5)	Adjuvant	CRT vs. RT alone	1994–2000	334	167	167	54/167 (32%)	66, NR	NR/167 & 150^c^/167	3 × 100 mg/m^2^	300	107/167 (64%)	110/167 (66%) & 132/167 (79%)
	7. Adelstein, 2003, Phase III (6)	Definitive	CRT vs. RT alone vs. split course CRT	1992–1999	295	97	87	52/87 (60%)	70, 2D	NR	3 × 100 mg/m^2^	300	74/87 (85%)	NR
	8. Forastiere, 2003, Phase III, RTOG 91-11 (7)	Definitive	concurrent CRT vs. sequential CRT vs. RT alone	1992–2000	547	182^a^	172	0/172 (0%)	70, NR	NR/172 & 157^c^/172	3 × 100 mg/m^2^	300	120/172 (70%)	NR & 160/172 (93%)
	9. Fountzilas, 2004, Phase III (15)	Definitive	CRT (cisplatin) vs. CRT (carboplatin) vs. RT alone	1995–1999	128	45	44	17/45 (38%)	70, 2D	NR/44 & 40/44	3 × 100 mg/m^2^	300	38/44 (86%)	NR

a From Forastiere et al. (14)

b Number of patients completing radiotherapy without any interruptions/number of patients started on radiotherapy & total number of patients completing radiotherapy as prescribed/number of patients started on radiotherapy

c *Bernier: defined as having received at least 60 Gy; Forastiere: defined as having received at least 95% of prescribed radiotherapy*.

*CRT, chemoradiotherapy; vs., versus; RT, radiotherapy; ITT, intention-to-treat population; OPC, oropharyngeal cancer; 2D, two-dimensional; 3D, three-dimensional; NR, not reported; N/A, not applicable*.

**Table 2:** Toxicity in prospective studies comparing chemoradiotherapy with weekly or three-weekly cisplatin vs. radiotherapy alone (4–7, 10–17).

**Concurrent cisplatin regimen**	**Study number**	**No. of pts. evaluable for acute toxicity**	**Gr. 3-4 acute toxicity during CRT**^**a**^																	**No. of toxic deaths (during CRT or within 30 days after completion)**	**30-day mortality (during CRT or within 30 days after completion), No. of cases**	**No. of pts. evaluable for late toxicity**	**Late toxicity**				
			**Anemia**	**Thrombocytopenia**	**Leukopenia**	**Neutropenia**	**Febrile neutropenia**	**Mucositis and/or stomatitis**	**Xerostomia**	**Dysphagia (pharynx/esophagus)**	**Nausea and/or vomiting**	**Weight loss**	**Laryngeal toxicity**	**Nephrotoxicity**	**Neurotoxicity**	**Ototoxicity**	**Skin toxicity**	**Diarrhea**	**Infection**				**gr. 3-4 overall prevalence^**e**^**	**gr. 1-2 xerostomia**	**gr. 3-4 xerostomia**	**gr. 3-4 dysphagia**	**gr. 3-4 subcutaneous fibrosis**
Weekly low-dose	**1**. (10)	39	3%	-	-	10%	-	21%	-	-	23%	21%	-	0%	-	-	-	-	-	-	-	30^c^	6^c^	-	-	-	3^c^
	**2**. (11)	77	-	-	-	-	-	-	-	-	-	-	-	-	-	-	-	-	-	0	-	-	-	-	-	-	-
	**3**. (12)	149	-	-	-	-	-	-	-	-	3%	-	4%	1%	1%	-	-	0%	3%	3	-	-	-	-	-	-	-
	**4**. (13)	65	-	-	-	-	9%	35%	-	-	-	-	-	-	-	-	23%	-	-	2	2	-	-	-	-	-	-
Three-weekly high-dose	**5**. (4)	204	3%	-	-	-	-	30%	2%	25%	20%	-	3%	2%	5%	-	7%	1%	6%	2	-	201	20%	55%^f^	3%	7%	1%
	**6**. (5)	167	-	-	16%	13%	-	41%	14%	12%	12%	-	1%	-	-	-	1%^b^	-	-	-	-	-	-	-	-	-	-
	**7**. (6)	87	20%	3%	46%	-	-	49%	-	-	17%	-	-	9%	-	-	8%	-	-	4	-	-	-	-	-	-	-
	**8**. (7)	171	-	-	-	-	-	43%	-	35%	20%	-	18%	4%^b^	5%	-	7%	-	4%	9	9	157^d^	-	67%^d^	6%^d^	16%^d^	6%^d^
	**9**. (15)	44	2%	4%	21%	9%	-	34%	0%	5%	23%	16%	0%	-	0%	-	2%	0%	5%	1	3	-	-	-	-	-	-

a *Adelstein: grade III-V acute toxicity*.

b *Forastiere: renal or genitourinary toxicities; Bernier: skin and connective tissue fibrosis*.

c Data from Bachaud et al. (16)

d Data from Forastiere et al. (14)

e On a per-patient basis (this information was not provided in the Bachaud et al. trial)

f *Data from Cooper et al., 2012, related to a population of 193 patients eligible for late toxicity assessment (17)*.

No., number; pts., patients; gr., grade; CRT, chemoradiotherapy

**Table 3:** Outcomes in prospective studies comparing chemoradiotherapy with weekly or three-weekly cisplatin vs. radiotherapy alone (4–7, 10–13, 15–17).

**Concurrent cisplatin regimen**	**Study number**	**Response rate**			**Number of patients eligible for survival analysis**	**Disease-free survival**		**Locoregional control**		**Distant control**		**Overall survival**		**Median follow-up (months): all patients/patients alive**
		**Number of patients evaluable for response**	**overall response**	**complete response**		**2-year**	**5-year**	**2-year**	**5-year**	**2-year**	**5-year**	**2-year**	**5-year**	
Weekly low-dose	1.^a^ (10)	N/A	N/A	N/A	39	68%	45%	84%	70%	73%	58%	72%	36%	36^d^^,^^e^ ns
	2. (11)	77	-	81%	77	47% (PFS)^b^	-	-	-	-	-	66%^b^	-	-/22
	3. (12)	149	79%	40%	149	23% (FFS)^b^	16% (FFS)^b^	-	-	-	-	31%^b^	16%^b^	62/-
	4. (13)	-	-	-	65	61%	39%	62%	49%	-	-	71%	56%	-/48
Three-weekly high-dose	5. (4)	N/A	N/A	N/A	206	53%^b^	35%^b^	82%	81%^b^	84%^b^^,^^c^	80%^b^^,^^c^	63%^b^	45%^b^	-/46^d^
	6. (5)	N/A	N/A	N/A	167	65% (PFS)^b^	47% (PFS)	84%^b^	82%	-	79%	73%^b^	53%^b^	61 ns
	7. (6)	87	-	40%	87	-	-	-	-	-	-	41%^b^	26%^b^	-/41^d^
	8. (7)	172	-	90%	172	61%	36%	78%	74%^b^	92%	88%	74%	54%	-/46^d^
	9. (15)	39	82%	51%	45	61% (TTP)^b^	49% (TTP)^b^	-	-	-	-	53%^b^	42%^b^	60^d^/-

a *Data from Bachaud et al. (16)*.

b *Values approximated from Kaplan-Meier survival curves*.

c *Data from Cooper et al. (17)*.

d *For all study arms*.

e *Mean value from Bachaud et al. (10)*.

*Abbreviations: N/A, not applicable; PFS, progression-free survival; FFS, failure-free survival; TTP, time-to-progression; ns, not specified (all patients/patients alive)*.

**Table 4:** Characteristics of prospective studies comparing chemoradiotherapy with weekly vs. three-weekly cisplatin (18, 19).

**Study number, author, year, study design**	**Therapy intent**	**Study arms**	**Inclusion period**	**Number of patients**				**Conventional radiotherapy**		**Concurrent cisplatin**			
				**ITT for the entire study population**	**ITT for cisplatin arm**	**Started on cisplatin therapy**	**OPC**	**Planned dose to the primary (Gy), technique**	**Radiotherapy compliance^**a**^**	**Planned schedule**	**Planned cumulative dose (mg/m^**2**^)**	**Patients receiving all planned cycles**	**Patients receiving at least 200 mg/m^**2**^ & at least 2 cycles**
9. Tsan, 2012, Phase III (18)	Adjuvant	Weekly low-dose cisplatin	2008–2010	55	NR	24	0/24 (0%)	66, NR	NR/24 & 22^b^/24	7 × 40 mg/m^2^	280	NR	15/24 (63%) & N/A
		Three-weekly high-dose cisplatin			NR	26	0/26 (0%)	66, NR	NR/26 & 24^b^/26	3 × 100 mg/m^2^	300	NR	23/26 (88%) & NR
10. Noronha, 2017, Phase III (19)	Adjuvant (93%) and definitive	Weekly low-dose cisplatin	2013–2017	300	150	148	2/146 (1%)	60 or 70, 2D	115/148 & 139/148	6–7 × 30 mg/m^2^	180–210	NR	NR
		Three-weekly high-dose cisplatin			150	148	3/148 (2%)	60 or 70, 2D	113/148 & 143/148	3 × 100 mg/m^2^	300	85/148 (58%)^c^	NR

a *Number of patients completing radiotherapy without any interruptions/number of patients started on radiotherapy & total number of patients completing radiotherapy as prescribed/number of patients started on radiotherapy*.

b *Defined as having received at least 60 Gy*.

c *However, 35/148 patients received two cycles, because radiotherapy was completed before the planned third dose*.

*Abbreviations: ITT, intention-to-treat population; OPC, oropharyngeal cancer; NR, not reported; 2D, two-dimensional; N/A, not applicable*.

**Table 5:** Toxicity in prospective studies comparing chemoradiotherapy with weekly vs. three-weekly cisplatin (18, 19).

**Study number**	**Study arm**	**No. of pts. evaluable for acute toxicity**	**Gr. 3-4 acute toxicity during CRT**^**a**^																	**Toxic deaths (during CRT or within 30 days after completion)**	**30-day mortality (during CRT or within 30 days after completion)**	**No. of pts. evaluable for late toxicity**	**Late toxicity**				
			**Anemia**	**Thrombocytopenia**	**Leukopenia**	**Neutropenia**	**Febrile neutropenia**	**Mucositis and/or stomatitis**	**Xerostomia**	**Dysphagia (pharynx/esophagus)**	**Nausea and/or vomiting**	**Weight loss**	**laryngeal toxicity**	**Nephrotoxicity**	**Neurotoxicity**	**Ototoxicity**	**Skin toxicity**	**Diarrhea**	**Infection**				**gr. 3-4 overall prevalence^**c**^**	**gr. 1-2 xerostomia**	**gr. 3-4 xerostomia**	**gr. 3-4 dysphagia**	**gr. 3-4 subcutaneous fibrosis**
9. (18)	Weekly	24	4%	0%	13%	4%	-	96%^a^^,^^b^	-	54%	21%	-	4%	0%	-	0%	8%	-	-	-	-	-	-	-	-	-	-
	3-weekly	26	4%	0%	0%	0%	0%	73%^a^^,^^b^	-	54%	12%	-	12%	0%	-	0%	8%	-	-	-	-	-	-	-	-	-	-
10. (19)	Weekly	148	2%	3%	3%^a^	1%^a^	1%^a^	16%	0%	43%	1%^a^	1%	9%	0%	0%	5%^a^	7%	1%	21%^a^	0	-	116	10%	54%^d^	1%	3%	0%
	3-weekly	149	5%	2%	16%^a^	13%^a^	6%^a^	18%	1%	39%	7%^a^	0%	9%	0%	0%	13%^a^	8%	5%	34%^a^	0	-	126	13%	53%	1%	4%	2%

a *Significant differences*.

b *According to personal communication*.

c *On a per-patient basis*.

d *Noronha: grade 2 late toxicity*.

*No., number; pts., patients; gr., grade; CRT, chemoradiotherapy*.

**Table 6:** Outcomes in prospective studies comparing chemoradiotherapy with weekly vs. three-weekly cisplatin (18, 19).

**Study number**	**Study arm**	**Response rate**			**Number of patients eligible for survival analysis**	**Disease-free survival**		**Locoregional control**		**Distant control**		**Overall survival**		**Median follow-up (months): all patients/patients alive**
		**Number of patients evaluable for response**	**overall response**	**complete response**		**2-year**	**5-year**	**2-year**	**5-year**	**2-year**	**5-year**	**2-year**	**5-year**	
9. (18)	weekly	N/A	N/A	N/A	24	-	-	60%^a^	-	-	-	72%^a^	-	12 ns
	3-weekly	N/A	N/A	N/A	26	-	-	57%^a^	-	-	-	72%^a^	-	12 ns
10. (19)	weekly	NR	NR	NR	150	48% (PFS)^a^	-	58%^a^	-	-	-	57%^a^	-	NR/22
	3-weekly	NR	NR	NR	150	55% (PFS)^a^	-	73%^a^	-	-	-	60%^a^	-	NR/22

a Values approximated from Kaplan-Meier survival curves

*N/A, not applicable; NR, not reported; PFS, progression-free survival; ns, not specified (all patients/patients alive)*.

### Post-operative Conventional Chemoradiotherapy

Between 1984 and 1988, Bachaud et al. randomized 88 patients to receive irradiation either alone or combined with weekly low-dose cisplatin. The enrolment was conditioned on histological findings of extracapsular extension of tumor in the affected lymph nodes, but not on infiltration of surgical margins. When tumor-free margins of at least 5 mm were attained, the dose to the primary site was 54 Gy, otherwise it was escalated to 65–70 Gy. At 5 years, overall survival (13 vs. 36%), disease-free survival (23 vs. 45%), and locoregional control (55 vs. 70%) were significantly higher in the chemoradiotherapy group, while the advantage in distant control rate was only numerical (49 vs. 58%). These improvements came at the cost of increased acute toxicity, consisting above all of weight loss, mucositis, nausea and/or vomiting, and myelosuppression. Grade 3–4 adverse events thus occurred in 16 and 41% of patients treated with radiotherapy and chemoradiotherapy, respectively, being only partially translated into long-term severe complications (15 vs. 20%) (10, 16).

Exploring three-weekly cisplatin, the Radiation Therapy Oncology Group (RTOG) 9501 and the European Organization for Research and Treatment of Cancer (EORTC) 22931 trials, were conducted and published about one decade later, relative to the study by Bachaud et al. Both RTOG 9501 and EORTC 22931 focused on high-risk patient groups. Most importantly, tumor specimens were characterized by the presence of extracapsular spread and/or positive margins which, in the case of EORTC 22931, also encompassed close margins up to 5 mm. The intention-to-treat populations consisted of 459 and 334 subjects in RTOG 9501 and EORTC 22931, respectively. In RTOG 9501, three-weekly cisplatin was associated with a significant prolongation of 5-year locoregional control (68 vs. 81%) and disease-free survival (25 vs. 35%), but without significant overall survival benefit (37 vs. 45%). In the EORTC study, the Kaplan-Meier estimates of all these three parameters were statistically improved (locoregional control: 69 vs. 82%; progression-free survival: 36 vs. 47%, overall survival: 40 vs. 53%). Systemic treatment with cisplatin had no meaningful impact on the risk of distant metastasis development in either study; five-year cumulative incidence ranged between 20 and 25% regardless of study arm. As expected, severe acute adverse events occurred more commonly in cisplatin-treated patients with mucositis rates being 18 vs. 30% and 21 vs. 41% in RTOG 9501 and EORTC 22931, respectively. Further chemotherapy-related side effects were mostly of hematological and gastrointestinal origin. Not clearly affected by systemic treatment, severe late toxicity ranged between 20 and 40% (4, 5, 17).

Looking at the three randomized trials together, there is a clear shortage of patients treated with weekly cisplatin under controlled clinical conditions. The high-dose cisplatin regimen was tested in a cohort almost 10 times larger. As to efficacy, the three-weekly regimen, again, numerically outperformed its competitor, though we cannot exclude the possible influence of stage migration reflected by the higher rate of distant failures in the earlier, low-dose cisplatin trial. In this respect, advances in diagnostics might have prevented some patients with clinically silent distant metastases from participation in the two subsequent studies with the three-weekly schedule. Further, appeals to low-dose cisplatin often rest on assuming its better toxicity profile. Unfortunately, between-trial comparisons of side-effects are confounded by incomplete and selective reporting, and evaluation of a number of side effects relies in part on each physician's expertise. Nevertheless, paying attention to patient's compliance might give us some important clues, because treatment toxicity plays a role in decreasing adherence to a given regimen. Bachaud et al. reported that only 59% of the study population could receive all planned cycles of cisplatin. The two studies on three-weekly high-dose cisplatin corroborated these findings with figures slightly above 60%. However, only a rigorous randomized trial could bring the ultimate vindication addressing all key aspects of the weekly vs. three-weekly schedules.

In 2012, Tsan et al. published the results of a small phase III trial randomly assigning 55 patients to one or the other concurrent cisplatin regimens. Both groups received the same mean doses of chemotherapy and radiotherapy, but significantly more patients could tolerate cumulative doses of at least 200 mg/m^2^ cisplatin in the high-dose arm (18). This threshold seems to be important, since mounting evidence suggests sufficient therapeutic effect when such an exposition to cisplatin is met (21–23). Besides that, despite lower cumulative doses, the weekly regimen produced more acute toxicity, particularly severe mucositis. Due to a median follow-up of only 12 months, the overall survival results remain preliminary with the following rates at 1 year: 79.3% with the three-weekly high-dose regimen vs. 71.6% with weekly cisplatin (*p* = 0.978) (18).

Another stream of evidence bolstering the three-weekly regimen came recently from a large randomized trial from India with 300 patients (19). The design was similar to the previous study except for the following major differences: (1) the investigators used a lower planned cumulative dose of weekly cisplatin (6–7 × 30 vs. 7 × 40 mg/m^2^ in the Tsan et al. trial) which might have compromised the comparison with 3 × 100 mg/m^2^ of three-weekly cisplatin; (2) patients could be treated both in the adjuvant and the definitive setting, although in the end, 93% belonged to the former group; and (3) all major SCCHN subsites (oral cavity, oropharynx, hypopharynx, larynx) and also patients with cervical lymphadenopathy of unknown primary could enter the study, although in the end, 87% subjects had oral cavity cancer, while Tsan et al. focused exclusively on oral cavity cancer (18, 19).

It is also of interest that in addition to positive surgical margins, the Indian trial accommodated cases with close margins (≤ 5 mm) which, as noted above, might steer the outcome. After a median follow-up of 22 months, the primary endpoint, estimated cumulative 2-year locoregional control, was improved by 14.6% in the three-weekly cohort (58.5 vs. 73.1%, *p* = 0.014). The resulting gains in median progression-free survival (17.7 vs. 28.6 months) and overall survival (39.5 months vs. not reached) fell short of statistical significance. The enhanced efficacy of the three-weekly regimen, albeit possibly influenced by the difference in cumulative doses, was offset by a higher incidence of acute adverse events (71.6 vs. 84.6%, *p* = 0.006), specifically in terms of vomiting, infection, hearing disturbance, hyponatremia, and myelosuppression. Occurring at a rate between 10 and 14%, severe chronic toxicity did not appear to be affected by the study medication. Remarkably, there were no significant differences between the two arms of the study in terms of treatment completion and compliance to the therapy (*p* = 0.1). Only 13.3% of patients did not receive the third cycle in the high-dose three-weekly arm for reasons of toxicity or patient refusal, while the 7th cycle in the low-dose weekly arm could not be given in 9.3% for toxicity reasons. Importantly, the number of administered chemotherapy cycles was influenced by the fact that 60 Gy of radiotherapy were planned in the prevailing adjuvant setting and the resulting six-week course usually finished before the last scheduled date of chemotherapy. Consequently, not more than two thirds of those treated with high-dose cisplatin could receive all three cycles and only slightly more of those allocated to the low-dose arm could benefit from all 7 cycles (19).

In summary, admitting a more rigorous scientific design of randomized comparative studies is still needed, post-operative use of three-weekly high-dose cisplatin given concurrently with conventionally fractionated radiotherapy in LA-SCCHN seems unassailable.

### Definitive Conventional Chemoradiotherapy

Three randomized trials with altogether 746 patients in the intention-to-treat population were conducted to evaluate the benefit of concurrent weekly low-dose cisplatin added to definitive conventional radiotherapy. In the first, Eastern Cooperative Oncology Group (E2382) study from Quon and co-workers, the accrual period began in 1982, about 20 years prior to the start of the latter two studies, authored by Ghosh-Laskar et al. and by Sharma et al. Between these three trials, there was a clear difference in the chosen target cumulative dose of cisplatin (7 × 20 vs. 7–8 × 30 vs. 7 × 40 mg/m^2^) which undoubtedly impacted on the observed outcomes. Quon et al. failed to show a meaningful improvement in the median failure-free survival, and the median overall survival was even numerically lower in those treated with combined therapy (13.3 vs. 11.8 months). Another reason for disappointment stemmed from significantly higher acute (nausea and/or vomiting, neurologic, renal, and haematologic), but also chronic (esophageal and laryngeal) toxicities elicited by weekly cisplatin (12). The three-arm trial performed by Ghosh-Laskar et al. was underpowered and had to be prematurely terminated after accruing 199 patients out of 750 planned enrolments. Despite a small improvement in locoregional control (*p* = 0.049), the target cumulative dose of 210–240 mg/m^2^ did not translate into overall survival advantage with 5-year rates being 36% in the radiotherapy alone arm vs. 56% in the combined modality arm (*p* = 0.112). Severe acute mucositis, but not skin toxicity was more common in the altered fractionation and chemoradiotherapy cohorts. Incidence of distant metastases and late toxicity did not differ among treatment groups (13). Relative to the E2382 study, Sharma et al. doubled the cumulative dose which apparently paid off. Echoed by a clear gain in median overall survival (27 months vs. no reached, *p* = 0.02), the complete response rate rose from 67.1 to 80.5% (*p* = 0.04). The increased severe acute toxicity rested at an acceptable 40% and was accompanied by a high adherence rate to cisplatin. No data on late side effects were reported (11).

Another three studies this time exploring the three-weekly regimen enrolled altogether 970 patients during the 1990s. Planned doses of chemotherapy (3 × 100 mg/m^2^ cisplatin) and radiotherapy (70 Gy) were set firmly, but were difficult to fulfill, especially with respect to cisplatin. Up to 30% of patients did not receive all planned cycles. Adelstein et al. and Fountzilas et al. showed a clear prolongation of median overall survival (from 12.6 to 19.1 months and from 12.2 to 48.6 months, respectively) accompanied by an increase in severe acute adverse events, notably hematological toxicity and nausea and/or vomiting, in the treatment arm with cisplatin. All grade 3-4 acute toxicities were as high as 85% in the study by Adelstein et al.; data on late effects are not publicly available. Contrary to expectation, compliance with all three cycles of cisplatin remained high at about 85% (6, 15). Concerning the third trial, Forastiere et al. set out to determine the value and optimal timing of chemotherapy as an adjunct to radiotherapy but strictly in patients with glottic and supraglottic larynx cancer. Compared with radiotherapy alone, concurrent three-weekly cisplatin resulted in a significantly better larynx preservation, locoregional control, and disease-free survival even at 10-years. Adherence to treatment was slightly inferior than in the previous two trials. Seventy percent of patients received all three concomitant cisplatin doses. From long-term view, there was also a trend toward improvement in distant control (from 76 to 84%), which was, however, not the case in the study reported by Adelstein et al. with rates about 80% across all treatment cohorts (6, 7).

We have learned from the interpretation of overall survival in the study by Forastiere et al. how important it is to report long-term results in such cases. The first paper from 2003, estimating 5-year overall survival after a median follow-up among survivors of 3.8 years, came to almost identical figures (about 55%) across all three treatment cohorts, i.e., radiotherapy alone, concomitant chemoradiotherapy, and induction chemotherapy followed by radiotherapy. Alarmingly, as published in 2013, at a median follow-up of 10.8 years, the survival curves started dissociating after about 4.5 years from randomization. This updated publication suggested a worse outcome in the concomitant compared with induction chemotherapy arm (*p* = 0.08), which could not be attributed to larynx cancer or the treatment itself. Although no significant differences in the 10-year cumulative rates of grade 3-5 late toxicities were detected (30–38%), it has been recognized, despite the extraordinary effort of the investigators to gather meaningful late toxicity data from this cooperative, multi-institutional study, that the results are inadequate, and the difference in survival reflects indeed an increase in delayed adverse events (7, 14). Radiation technique represents another important variable. As a general rule, two dimensional and three dimensional treatment planning has been linked to severe late side effects, which are rather uncommon in the current era of image-guided intensity-modulated radiotherapy (IMRT) with or without cisplatin (24). Anyway, the increase of deaths from non-cancer related causes is worrisome and places an even greater onus on accurate and complete reporting of studies.

With the debatable exception of the Indian trial mentioned in Post-operative Conventional Chemoradiotherapy, no other prospective studies compared low-dose weekly with high-dose three-weekly concurrent cisplatin in the definitive setting.

## Evidence from a Meta-Analysis of 59 Trials

In two recent papers we analyzed aggregate data from altogether 59 prospective trials to take up the comparison between the two concurrent cisplatin regimens (8, 9). Consisting of three separate meta-analyses as explained below, our work offers further insight into the conundrum of low-dose vs. high-dose cisplatin. Within the context of available large phase III clinical data favoring more or less the three-weekly approach, the basic idea was to figure out which one of the following two hypotheses is suitable for adoption by the medical community:

- The low-dose regimen has more potent anti-tumor properties than its competitor. Ergo, low-dose cisplatin should be considered the new non-surgical and/or adjuvant standard of care in the clinical scenario of LA-SCCHN.
- The low-dose regimen does not outperform high-dose cisplatin. Hence, high-dose cisplatin should remain the standard of care, while more research is warranted on the weekly protocol.

Afterwards, the key step was to define the appropriate primary objective. In this respect, overall survival is generally accepted as a reliable outcome endpoint. Caution needs to be advised when interpreting other measures, which may rest on weak evidence. For instance, different author groups use different criteria to estimate progression-free or disease-free survival as well as locoregional or distant control rates. The resulting heterogeneity impedes data merging and running a proper meta-analysis. Correspondingly, an inter-trial evaluation of adverse events and compliance has been confounded by an incomplete and selective reporting. Moreover, inherent issues in toxicity data collecting include the distinct aim of the study, a subjective influence of each physician on the assessment, individual variations in the emergence of side effects which may not always coincide with their measurement, and the existence of different toxicity scales and guidelines. As an illustration, the highest rate of severe acute laryngeal toxicity (18%) was noted in the Forastiere et al. study, which focused specifically on laryngeal preservation. Considering all these aspects, overall survival was chosen as the reference endpoint in our meta-analysis.

**Figure 1:**
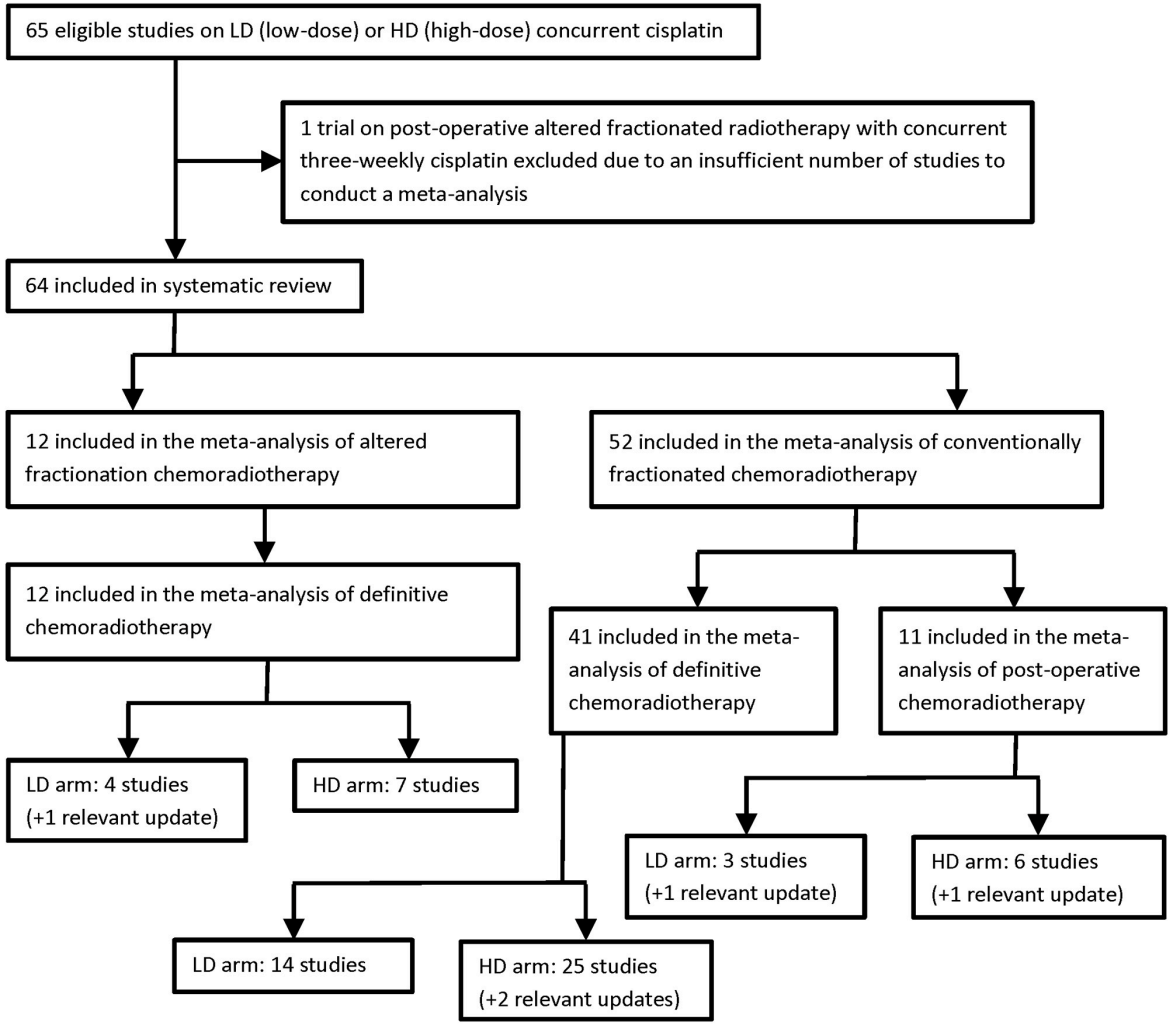
Flow chart of study distribution into three separate meta-analyses (8, 9).

To reflect the specific biological effects of different radiotherapy fractionation schedules and to separate the adjuvant from the definitive treatment intents, three meta-analyses were carried out, one for postoperative conventional chemoradiotherapy, one for definitive conventional chemoradiotherapy, and one for definitive altered fractionation chemoradiotherapy. Published data were insufficient to run a meta-analysis in the setting of postoperative altered fractionation chemoradiotherapy. The paucity of data can be explained by the fact that relative to conventional fractionation the altered scheme does not seem to bring any survival advantage after surgical resection and is even associated with a higher rate of severe acute mucositis (25). Selection criteria and the flow diagram of study distribution are detailed in Table 7 and Figure 1, respectively. Other methodological aspects will not be presented here, as they have been exhaustively explained in both publications. The following subsections will cover the most important results in terms of efficacy, toxicity, and compliance. They set out to provide a summarizing interpretation with implications for clinical practice.

**Table 7:** Selection criteria pertinent to the composite meta-analysis of weekly low-dose vs. three-weekly high-dose concurrent cisplatin (8, 9).

	**Key inclusion criteria**	**Key exclusion criteria**
1.	Full-text articles published up to December 1, 2015	Other language than English
2.	Prospective studies	Updates and additional investigations of previously reported patient populations with no new relevant data
3.	Locally advanced squamous cell carcinoma of the head and neck (stage III-IVB)	No standard reporting of efficacy and/or toxicity
4.	Treatment-naive tumors	>50% of patients had cancer of the nasopharynx or salivary glands and/or recurrent tumors
5.	Concurrent chemoradiotherapy either in the definitive or adjuvant settings	>25% had incomplete specification of treatment schedule
6.	Separate evaluation of conventional and altered fractionation radiotherapies	>25% treated with induction chemotherapy
7.	High-dose protocol during conventional fractionation: 100 mg/m^2^ cisplatin on days 1, 22, and 43 (alternatively 2, 23, 44)	>25% treated using different time intervals, doses, or routes of application of cisplatin
8.	High-dose protocol during altered fractionation: 100 mg/m^2^ cisplatin on days 1 and 22 (alternatively 1 and 28)	>25% treated using alternative radiotherapy protocols
9.	Low-dose protocol during conventional fractionation: ≤ 50 mg/m^2^ cisplatin weekly at least 6x	>25% had cisplatin combined with other drugs
10.	Low-dose protocol during altered fractionation: ≤ 50 mg/m^2^ cisplatin weekly at least 4x	>25% had chemoradiotherapy in hyperthermia

### Efficacy

**Figure 2:**
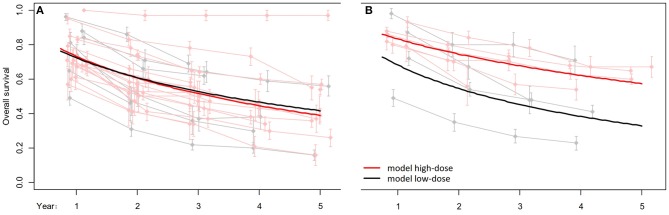
Overall survival analysis comparing high-dose vs. low-dose cisplatin given concurrently with conventional **(A)** and altered fractionation **(B)** radiotherapy in the definitive setting. Reprinted in part from Szturz et al. (8). Copyright © 2017, with permission from AlphaMed Press, and from Szturz et al. (9). Copyright © 2018, with permission from Elsevier.

In the frame of conventionally fractionated radiotherapy, no statistically significant difference in overall survival was observed between low-dose weekly and high-dose three-weekly cisplatin (Table 8, Figure 2). This was true for both the adjuvant (*p* = 0.5345) and the definitive (*p* = 0.8519) treatment settings. But do overlapping survival curves automatically mean equipotency of the respective regimens? To answer this, several aspects have to be taken into account. First, it should be kept in mind that the results originate from a meta-analysis, and not from a randomized clinical trial. Our meta-analysis comprised data from trials that were typically uncontrolled or did not compare low- vs. high-dose cisplatin schedules. Consequently, the pooled data related to patient groups that were not intended to be compared prospectively, leading to an increased likelihood of selection, confounding, and reporting biases. In other words, the two pooled patient populations treated with either low- or high-dose cisplatin were not selected according to exactly the same stratification criteria. Second, there were discrepancies in treatment adherence expressed as proportion of patients who received all planned cycles of chemotherapy. In both the adjuvant and definitive settings, compliance was worse with high-dose cisplatin (71 vs. 64% and 88 vs. 71%, respectively), although this difference reached statistical significance only in the latter setting (*p* = 0.5747 and *p* = 0.0017, respectively). Lower compliance signifies that the mean cumulative dose in a given patient cohort is also decreased. Thus, it may be speculated that even with administration of a lower amount of cisplatin correlating with lower compliance, the high-dose regimen managed to maintain a sound antitumor activity comparable with its low-dose counterpart. If this is true, then we will be able to show an improvement in overall survival with a lower planned cumulative dose of three-weekly cisplatin, where compliance can be expected to rise.

**Table 8:** Model-based estimates of overall survival according to the three meta-analyses (8, 9).

**Overall survival at…**	**Conventional fractionation**			
	**Definitive treatment**		**Post-operative treatment**	
	**Weekly cisplatin (%)**	**Three-weekly cisplatin (%)**	**Weekly cisplatin (%)**	**Three-weekly cisplatin (%)**
1-year	72	73	75	79
2-year	61	61	66	69
3-year	53	52	60	62
4-year	47	45	55	56
5-year	41	39	51	51
**Overall survival at…**	**Altered fractionation**			
	**Definitive treatment**		**Post-operative treatment**	
	**Weekly cisplatin (%)**	**Three-weekly cisplatin (%)**		
1-year	68^a^	83^a^	Meta-analysis was not conducted due to an insufficient number of eligible studies	
2-year	55^a^	74^a^		
3-year	45^a^	68^a^		
4-year	38^a^	62^a^		
5-year	33^a^	57^a^		

a *Significant differences*.

And this is indeed exactly what happened in the altered fractionation model. Herein, there was a clear survival advantage with two cycles of high-dose cisplatin over the low-dose applications (*p* = 0.0185; Table 8, Figure 2). This is in line with compliance outcomes favoring also the high-dose regimen (71 vs. 92%, *p* = 0.0353). So numerically, notwithstanding the use of different radiotherapy techniques, we see a much better compliance in patients treated with two (92%) in comparison with three (64–71%) cycles of high-dose cisplatin, while adherence to low-dose cisplatin remains more consistent (71–88%). Pertaining to definitive chemoradiotherapy, response rates were similar between low- and high-dose arms in both conventional and altered fractionation models (please see for further details both source publications).

However, proponents of low-dose cisplatin would probably argue against these conclusions. Their interpretation would presumably rest on the notion that if low-dose cisplatin exhibits a comparable survival outcome as the high-dose schedule, both approaches must be equally effective and can therefore be applied interchangeably to clinical practice. At this point it should be emphasized once again that data from randomized clinical trials, as debated above, are not sufficient to prioritize the low-dose over the high-dose protocol, and the absence of a survival benefit in our composite meta-analysis perfectly affirms this observation. Further support of our arguments relates to the fluctuations in treatment adherence and the results in the altered fractionation chemoradiotherapy.

### Toxicity

In each of the three meta-analyses, statistically significant differences were detected in several acute toxicity parameters, but not every case is supported by a sufficient number of source studies. Although weekly cisplatin given concurrently with postoperative conventional radiotherapy exhibited higher rate of grade 3-4 dysphagia and weight loss than three-weekly cisplatin, each of these two findings is based on only one study in the weekly arm, which is also true for weight loss in the three-weekly arm (10, 18, 26). In addition, in the one study on weekly cisplatin reporting severe dysphagia, patients did not receive hydration routinely (18). More convincingly, in definitive conventional chemoradiation, the three-weekly protocol clearly showed higher haematotoxicity (*p* = 0.0083 for leukopenia and *p* = 0.0024 for neutropenia), nausea and/or vomiting (*p* < 0.0001), and nephrotoxicity (*p* = 0.0099), thus typically cisplatin-related adverse events. But here again, the negative contribution of the third high-dose cisplatin dose to the observed acute toxicity can be hypothesized. This explanation may in fact be plausible. In the altered fractionation setting, where the target cumulative dose oscillates around 200 mg/m^2^, corresponding with two high-dose cisplatin doses, it was the weekly regimen that was associated with an increased burden of severe acute side effects. The difference was particularly apparent for mucositis and/or stomatitis (*p* = 0.0202), but also concerned constipation (*p* = 0.0066). Yet caution is required once more, because the data on constipation in the weekly regimen was based only on one study (27).

Acute toxicity closely allies with mortality, where two variables have been distinguished. One is represented by grade 5 toxicity, defined as toxic death during chemotherapy or within 30 days after its completion, the second by 30-day mortality. While both parameters did not seem to be affected by different cisplatin scheduling during conventional radiotherapy, low-dose weekly cisplatin induced significantly more grade 5 adverse events and a higher 30-day mortality than high-dose three-weekly cisplatin during altered fractionation radiotherapy. It is tempting to point out the absence of the third cisplatin cycle as being responsible for the apparently better results in the three-weekly altered fractionation arm. On the other hand, the decisive factor might have also been the altered radiotherapy scheme itself because of the variety of existing fractionation patterns which could not be utterly eliminated in the meta-analysis leading to a possible stratification bias. Moreover, altered fractionation is known to be more powerful than conventional fractionation (28).

For any treatment with curative intent, accurate recognition, systematic monitoring, and meticulous reporting of late adverse events are of paramount importance. The 2013 update on the study by Forastiere et al. includes an illustrative example to that (14). However, despite the major implications on quality of life of long-term cancer survivors, chronic toxicity has often been underreported, and concerns about the reliability of toxicity data have been risen (29). Entailing difficulties for interpretation, most of the studies enrolled in our meta-analyses lack long-term follow-up information. Analogously to the previously mentioned mortality outcomes, the only statistical difference was found in altered fractionation chemoradiotherapy, particularly in severe late subcutaneous fibrosis, which was significantly more common in one trial of low-dose cisplatin compared with three studies on the high-dose regimen (*p* < 0.0001).

### Compliance

Treatment adherence constituted the third touchstone of our meta-analyses. It accounted partly for the observed differences in survival as well as reflected those in toxicity. We looked separately at the compliance to radiotherapy and chemotherapy. With respect to radiotherapy, both arms achieved comparable results irrespective of therapy intent or fractionation schedule. This seems logical, given the equal characteristics of radiotherapy in both arms of each of the respective meta-analyses, but reporting this is important to show that the disparities in compliance were primarily driven by systemic therapy. The only exception was a trend toward worse radiotherapy compliance in the weekly altered fractionation arm in terms of completion as prescribed (*p* = 0.0659). However, this could be easily explained by the worse overall tolerance of that arm, because impaired compliance was also apparent in the analysis of cisplatin administration. In this respect, it should be remembered that due to the limited added value of chemotherapy in chemoradiotherapy protocols of SCCHN, it is the systemic treatment in the first place which is usually reduced or interrupted in the case of severe acute toxicity. Unplanned radiotherapy breaks were linked to significantly worse locoregional control which may decrease as much as by 1.2% for every day of interruption, and this does not seem to be compensated by the use of chemotherapy (30).

Summing up the topic, which has already been addressed in the previous sections, in the conventional chemoradiotherapy meta-analyses, the proportion of patients who received all prescribed chemotherapy cycles was lower in those treated with three-weekly cisplatin. In the postoperative setting, this relationship fell short of statistical significance (71 vs. 64%, *p* = 0.5747), but the influence of limited source data in the weekly arm, stemming from only two studies, cannot be ruled out. In the definitive setting, the difference was indisputable (88 vs. 71%, *p* = 0.0017), being backed up by a higher number of enrolled trials. In contrast, in the meta-analysis of altered fractionation it was the high-dose protocol reaching better adherence (*p* = 0.0353), which might be a consequence of the specific kinetic pattern of severe acute adverse events usually peaking at 4–5 weeks after chemoradiotherapy initiation, that is when two cycles of three-weekly cisplatin have already be delivered (31, 32).

The primary difference between cisplatin given concurrently with conventional and altered fractionation radiotherapies is the cumulative dose which tends to attain 300 and 200 mg/m^2^, respectively. Therefore, subtracting radiotherapy, it may be hypothesized that the lower cumulative dose, given in an appropriate schedule, may ultimately yield better outcomes due to better compliance and lower toxicity.

## Special Considerations

### Cumulative Dose

To continue on the subject of cumulative dose, we will mention several pivotal papers complemented by our own observations. In a systematic review, Strojan et al. constructed a model based on 6 phase III trials of definitive chemoradiotherapy. They demonstrated that regardless of the cisplatin schedule used (daily, weekly or three-weekly), escalating the dose of concurrent cisplatin in the range between 140 and 270 mg/m^2^ was significantly associated with overall survival (*p* = 0.027). For every 10 mg/m^2^ of cisplatin the magnitude of absolute benefit between combined modality treatment and radiotherapy alone rose by 2.2% (33). Wong et al. searched the Longitudinal Oncology Registry of Head and Neck Carcinoma (LORHAN) for newly diagnosed LA-SCCHN patients treated with chemoradiotherapy. In a cohort of 1,091 cases, higher cumulative doses of cisplatin were obtained in those treated with the high-dose three-weekly schedule than in those treated with the low-dose weekly cisplatin schedule (*p* < 0.001) and this had an impact on overall survival (34). Similar results were achieved in an unadjusted analysis of 2,901 patients using the population-based Veterans Affairs dataset (35). However, the outcomes of the latter two studies must be interpreted with caution because of their retrospective nature. It cannot be ruled out that patients in the low-dose weekly arm were less vigorous and more likely to have their treatment and survival compromised as suggested by adjusting for performance status of the veterans which eliminated the initially observed survival difference.

Other investigators have posited that a cumulative dose of 200 mg/m^2^ cisplatin produces an adequate anti-tumor effect in terms of overall survival. Their opinion resides on an indirect comparison with data stemming from randomized trials of alternative regimens in LA-SCCHN or nasopharyngeal cancer, on a retrospective observation of patients treated with weekly cisplatin, on a meta-analysis involving randomized trials comparing concomitant platinum- and platinum plus fluorouracil-based chemoradiation vs. radiotherapy alone, and on a recently published large randomized trial in which the median total cisplatin dose received in the standard high-dose arm was 200 mg/m^2^ (22, 23, 36, 37). Stronger evidence for this notion was conveyed by the RTOG 0129 phase III trial assigning patients to receive either two cycles of three-weekly high-dose (100 mg/m^2^) cisplatin concurrent with accelerated radiotherapy or three cycles of the same high-dose cisplatin with conventionally fractionated radiotherapy (38). Although no improvement in outcome was seen in one or the other patient cohort, subgroup testing according to the number of delivered cisplatin cycles and radiotherapy fractionation merits further attention. In the overall survival analysis, irrespective of fractionation, giving only one dose of 100 mg/m^2^ cisplatin was significantly worse than any other combination, but no advantage was seen with three over two cycles of cisplatin. Progression-free survival turned out alike. Disappointingly, cancer-specific survival curves by distant metastasis overlapped, regardless of chemotherapy or radiotherapy characteristics. However, one of the most intriguing results concerned locoregional control. Here, three cycles of cisplatin together with conventional radiotherapy yielded better outcome than one cycle (*p* = 0.049 for both fractionation schemes), but also than two cycles (*p* = 0.047 for altered fractionation and 0.11 for conventional radiotherapy) (21).

Taken together, a cumulative dose of at least 200 mg/m^2^ of cisplatin seems to ensure an adequate survival benefit in comparison with radiotherapy alone, but it is currently unknown whether a further increase brings additional survival prolongation or whether this is not offset by escalated toxicity responsible for an increment in non-cancer related deaths. On the other hand, good locoregional control, being the possible goal of cumulative doses around 300 mg/m^2^, is for sure one of the paramount factors in these patients. LA-SCCHN affects a region prone to visible disfigurement with far-reaching functional and esthetical aspects, and therefore reaching local/regional control legitimizes full-dose treatment. The cumulative cisplatin dose issue seems to play a different role in human papillomavirus (HPV)-positive oropharyngeal cancers, a disease entity with a rapid rise in incidence in western societies, with a much better prognosis, and for which we hope that less aggressive therapies might lead to at least similar outcome as presently obtained with the standard approach, but with less (acute and late) toxicity (39).

Researchers from Canada and Italy conducted a pooled analysis of 659 patients with stage III and IV oropharyngeal cancer, carcinoma of unknown primary, and laryngo-hypopharyngeal cancer who had been treated between 2000 and 2012 in two tertiary academic cancer centers with single-agent cisplatin during radiotherapy. All patients were treated with IMRT or three-dimensional conformal radiotherapy (3DRT) to a gross tumor dose of 60–70 Gy in 33–35 fractions over 6.5–7 weeks (2 Gy per fraction). Concurrent cisplatin regimens were either high-dose 100 mg/m^2^ three-weekly on days 1, 22, and 43 or low-dose 40 mg/m^2^ weekly for 7 weeks, the choice of which was based on institutional guidelines taking into account patient factors including Zubrod Performance Scale and comorbidities. Three-year overall survival for cisplatin <200 mg/m^2^, equal to 200 mg/m^2^, and above 200 mg/m^2^ subgroups were 52, 60, and 72% (*p* = 0.001) for the HPV-negative and 91, 90, and 91% (*p* = 0.30) for the HPV-positive patients. A multivariate analysis confirmed a survival benefit with cisplatin above 200 mg/m^2^ for HPV-negative patients (hazard ratio [HR] 0.5, 95% confidence interval [CI]: 0.3–0.7, *p* < 0.001) but not for HPV-positive patients (HR 0.6, 95% CI: 0.4–1.1, *p* = 0.104). There was a superior overall survival trend in the HPV-positive T4 or N3 high-risk subset (*N* = 107) with cisplatin above 200 mg/m^2^ (HR 0.5, 95% CI: 0.2–1.1, *P* = 0.07) (40). Of interest, the recently reported de-escalation trial RTOG 1016, using 2 cycles of 100 mg/m^2^ cisplatin with altered fractionation radiotherapy in patients with low- and intermediate risk HPV-positive oropharyngeal cancer, showed superior outcome vs. the same radiotherapy plus weekly cetuximab (an anti-epidermal growth factor receptor [EGFR] monoclonal antibody approved for this indication) (41).

### Two vs. Three Cycles

In the definitive setting, our composite meta-analysis favored concurrent altered fractionation radiotherapy with two cycles of high-dose cisplatin. Owing to different radiotherapy fractionation schedules, an interpretation of a direct comparison with three-cycles of high-dose cisplatin combined with conventional radiotherapy would be problematic. Nevertheless, a compromise solution using absolute values may be worth pursuing. In this respect, the former regimen achieved higher overall response rates (89 vs. 80%), complete response rates (74 vs. 60%), overall survival (5-year rates: 57 vs. 51%), and also compliance with all planned cycles of cisplatin (92 vs. 71%). Severe adverse events were numerically comparable except for two parameters. The first was acute dysphagia (40 vs. 26% in altered fractionation vs. conventional chemoradiotherapy, respectively), which is not a typically chemotherapy-induced side effect, but rather being related to differences in fractionation, and the second was late toxicity (43 vs. 14%, respectively), which is worth noticing, albeit that, as mentioned earlier, its reporting is often inaccurate and suffers from further biases.

Conclusions of such an indirect comparison are in line with the results from our individual meta-analyses. The only setting where a difference in overall survival was demonstrated was altered fractionation with high-dose cisplatin surmounting weekly regimens. Moreover, as alluded to above, it might have been the third cycle of high-dose cisplatin responsible for inferior compliance and greater toxicity in the definitive setting with conventional chemoradiotherapy. In addition, taking into account the mounting evidence on the significance of cumulative dose, the long-term RTOG 0129 trial data, and the recently reported outcome of RTOG 1016, it may follow that a minimum of two cycles of 100 mg/m^2^ cisplatin given concurrently during definitive (and probably also post-operative) radiotherapy provide the optimal drug exposition, when toxicity and compliance issues are taken into consideration. Nevertheless, it will remain unclear whether the prolonged survival observed in retrospective analyses in patients receiving a dose beyond the 200 mg/m^2^ is due to the higher dose itself or because of a better general condition, making it possible for them to receive an additional cisplatin dose.

### Clinical Practice Recommendations

The high rate of systemic and mucosal toxicities associated with high-dose cisplatin during radiotherapy led many trialists and clinicians to seek for alternative regimens with diminished treatment-related complications, improved compliance, and maintained anticancer activity. The theoretical background for weekly cisplatin has been further enriched by assumptions that compared with the high-dose schedule it has a superior capacity (1) to facilitate timely dose adjustments, (2) to enhance radiosensitization of the tumor, and (3) to lower costs and increase the feasibility by being able to administer this lower dose in the outpatient setting (8). Further support for its use have been the positive results obtained in patients with nasopharyngeal and uterine cervical cancers treated with low-dose cisplatin-based chemoradiotherapy (42, 43).

However, none of these claims have ever been based on sufficient clinical evidence, and extrapolations from other disease entities would have been unnecessary if a proper phase III trial had been available. In order to offer the best possible care outside clinical trials for the individual patient, taking into account what can be considered optimal standard chemoradiation, i.e., 100 mg/m^2^ given three times at three-week intervals concurrently with conventionally fractionated radiotherapy, three clinical situations may arise:

1. There are absolute contraindications to use cisplatin (e.g., poor performance status, renal failure, overt acquired immunodeficiency syndrome, pregnancy, or allergy to the agent) (44). In this scenario, both high- and low-dose regimens are excluded. In combination with definitive radiotherapy, some of the viable alternatives supported by randomized clinical research comprise carboplatin plus fluorouracil or cetuximab (45–47). Other options based on patient and disease characteristic include conventionally fractionated radiotherapy alone in both settings and altered fractionation radiotherapy, preferably hyperfractionation, in the definitive disease setting (28, 48).
2. There are relative contraindications to use cisplatin (e.g., dysfunctions of various organs) (44). This clinical situation represents the so-called gray zone in medical decision making. Here, if no clear guidelines for dose reductions exist, the relativity of the contraindications implies for some practitioners the need of treatment modifications, while the others might not permit any changes to the plan. We believe that under these circumstances lowering the peak concentration (which is an important determinant for acute toxicity) by either a prolonged infusion or dose reduction are defendable options (44, 49, 50). Alternatively, the options mentioned above in the subsection on what to use in case of absolute contraindications to cisplatin also hold for patients with relative contraindications. Again, patient and disease characteristics are crucial in decision making, and all aspects should be discussed in the multidisciplinary tumor board.
3. There are no contraindications to use cisplatin. The patient is in a good general condition, has no or few comorbidities and is willing to adhere to the treatment program. The standard approach in the definitive disease setting is to give two or three cycles of high-dose cisplatin during altered fractionation or conventional radiotherapy, respectively, which should be pursued whenever possible. In the adjuvant setting, only the latter radiotherapy fractionation is the current evidence-based option. In addition, there is an important role for the clinician, not only to stimulate patients to adhere to the treatment schedule, but also to give him/her maximal supportive care in order to make it tolerable for the patient. A minimum cumulative cisplatin dose of 200 mg/m^2^ should be aimed for and when conventional fractionation radiotherapy is used, the third cycle should only be given when toxicity permits.

## Conclusions

The landscape of SCCHN has been undergoing important epidemiologic transitions, which have notable impact on patient outcome, disease classification, and will probably also diversify treatment options. In economically developed countries, the role of chemoradiation with three-weekly high-dose cisplatin has been compromised by an ever growing number of HPV-positive oropharyngeal cancer cases, with 5-years survival rates even exceeding 80% in the more advanced disease settings (according to the 7th AJCC classification), which entails potential long term toxicities and therefore gives priority to de-escalation strategies (39, 51). However, this de-escalation approach has recently been brought into question by two large randomized trials (37, 41). Moreover, in the rest of the world, in which the majority of patients present with HPV-negative disease, facing a more unfavorable outcome, optimal use of platinum-based chemoradiation remains the cornerstone of the multi-modality management in LA-SCCHN.

The primary take-home message of this review is that repeatedly confirmed results of well-conducted, large, randomized trials should be respected unless proven otherwise. Second, the applicability of modern oncology research may be limited in many parts of the world, for which improvements in established treatment solutions are of potential value. And third, weekly low-dose cisplatin is not superior to the three-weekly high-dose regimen, which should therefore remain the standard of concomitant chemotherapy during external beam radiotherapy in LA-SCCHN.

In oncology, and this is certainly the case in head and neck oncology, there is an urgent need for new treatment modalities with high efficacy, low-toxicity, and ease of administration, tailored to the individual patient. Particularly modern immunotherapy supported by reliable predictive biomarkers has the greatest potential to replace or act as an add-on to present therapies. However, the downside is still its limited accessibility and affordability and currently also the lack of data in the locally advanced setting. Until this is resolved, the classical chemoradiotherapy will preserve its global relevance.

## Author Contributions

PS and JV drafted the manuscript; KW conducted the statistical analysis; DA and DV contributed to writing of the manuscript; NK, MT, KP, and VN contributed to the conception and reviewed the manuscript.

### Conflict of Interest Statement

PS: Honoraria received: Merck-Serono. NK: Consulting/advisory relationship: ONO, Bristol-Myers Squibb, Bayer, and Merck-Serono. Research funding: ONO, Bristol-Myers Squibb, Pfizer, AstraZeneca, and Roche. Honoraria received: ONO, Bristol-Myers Squibb, Bayer, Merck-Serono, and AstraZeneca. MT: Consulting/advisory relationship: Merck Sharp & Dohme, Bayer, AstraZeneca, Pfizer, Ono, Bristol-Myers Squibb. Research funding: Eisai, Boehringer Ingelheim, Novartis, NanoCarrier, Merck Sharp & Dohme, Ono. Honoraria received: Ono, Eisai, Bayer, Bristol-Myers Squibb. DV: Honoraria received: Sanofi, Accuray. JV: Consulting/advisory relationship in the last 3 years: Amgen, AstraZeneca, Boehringer Ingelheim, Innate Pharma, Merck Serono, Merck Sharp & Dome Corp, PCI Biotech, Synthon Biopharmaceuticals, Debiopharm, and wntResearch. Honoraria received: Merck-Serono, Sanofi, and BMS.

The remaining authors declare that the research was conducted in the absence of any commercial or financial relationships that could be construed as a potential conflict of interest.
